# A new extended single-switch high gain DC–DC boost converter for renewable energy applications

**DOI:** 10.1038/s41598-022-26660-7

**Published:** 2023-01-06

**Authors:** Arafa S. Mansour, Mohamed S. Zaky

**Affiliations:** 1 Electrical Engineering Department, Faculty of Engineering, Beni-Suef University, Beni-Suef, 62511 Egypt; 2 Department of Electrical Engineering, College of Engineering, Northern Border University, Arar, 1321 Saudi Arabia

**Keywords:** Electrical and electronic engineering, Solar energy

## Abstract

High-gain DC/DC converters are considered one of the most important components of green energy systems. Large numbers of these converters are used for increasing the voltage gain by using an extreme duty cycle. However, it increases losses and the cost, degrades the system performance, and hence obtains a low efficiency. In this article, a new design of a high-gain DC/DC boost converter is proposed. This converter has the potential to be used in low input voltage applications that need a high voltage gain such as systems powered by solar photovoltaic panels and fuel cells. The new topology is characterized by its simplicity of operation, high voltage gain, better efficiency, continuity of the input current, reduced number of inductors and capacitors, and can be extended to get higher gains. The converter structure, principle of operation, and design consideration of inductors and capacitors are presented in detail. Derivation of power losses and efficiency is presented. A laboratory prototype is implemented, and various experimental tests are given. The achievement of the suggested design is confirmed and compared with other recent high-gain converters.

## Introduction

The usage of renewable energy sources (RESs) is becoming more common and crucial as a result of the fast-rising demand for electrical energy and the expanding use and higher production costs of fossil fuel-based energy resources. One of the renewable energy sources, photovoltaic (PV) energy, has experienced rapid development during the past few decades. PV sources' output power and voltage are variable, nonlinear, and dependent on the surrounding temperature, solar radiation levels, and load values^1^. To connect the PV sources to the electricity grid and obtain the maximum energy possible from these sources under different operating conditions, high step-up voltage DC/DC converters with greater efficiency are used to emphasize the right tracking of the maximum power and lift the low PV voltage level to proper DC levels needed for inverters^2^. DC/DC boost converters are used for a variety of practical applications, which require high levels of DC voltage with small input current ripples^3^. One of the most important of these applications is RESs. The traditional DC/DC boost converter includes four components: a power switch, diode, input inductor, and output capacitor. It has a simple structure with a low price, and it is characterized by a non-pulsating input current in the continuous conduction mode (CCM) operation^4^. Nevertheless, the main disadvantages of this converter are extreme conduction losses, severe switching stresses, excessive input current ripples, and rising electromagnetic interference (EMI). Additionally, it is improper for applications that need raising voltage gains.

Many research efforts have been done to get further voltage gain without working at an extreme duty ratio and to remedy the abovementioned issues^5^. An interleaved DC/DC boost converter was introduced in^6^. It used two standard boost converters connected in parallel. The reliability of the interleaved converter based on the Markov standard was presented in^7^. To minimize the ripples of the input current, the switching losses and EMI levels, different PWM control schemes were introduced in^8^, and another control structure based on a look-up table was stated in^9^. However, they have problems with isolating gate signals and increasing the cost. Moreover, the voltage gain was identical to the conventional converters^10^. Interleaved boost converter based on a voltage doubler was used^11^. Another control method by combining alternating phase shift control and customary interleaving PWM control for the interleaved converter was proposed in^12^. A six-phase interleaved DC/DC converter with switched capacitor voltage multiplier cells was listed in^13^. These structures confirmed low current ripples, large voltage gain, and balancing of voltage capacitors. However, the main issues were the duty cycle restriction and rising cost. An assortment of DC/DC boost converters based on a switched capacitor was suggested in^14^ and a switched capacitor/inductor was hired in^15^. High voltage gain was satisfied, but they have large conduction losses, serious switching stresses, and circuit complexity. In^16^, a cascaded boost converter was used with high-voltage gain, and low switching losses, and current ripples. However, shortcomings were noted such as rising cost, decreased efficiency, and complexity of the control system.

Different topologies in the literature were introduced for realizing high-voltage gain such as coupled inductor/capacitor converter^17^, voltage-cl amped coupled inductor converter^18^, a standard boost in series with flyback converter^19^, and classic converter with a coupled inductor and a voltage lift cell^20^. Nevertheless, several issues like lowest efficiency, adding EMI levels, pulsing input current, high price, and complexity of the drive circuit are noted. To decrease the output diode voltage stress of the flyback cell and improve the efficacy, a coupled inductor boost combined flyback converter was suggested in^21^. However, a snubber circuit is needed to absorb the surges across the main power switch. A DC/DC converter that used voltage multiplier cells of capacitor-inductor-diode with a voltage lift circuit to boost the voltage gain was introduced in^22^. High voltage gain and low switches voltage stresses were accomplished. Unfortunately, because the main switch is connected in series with the input DC source, the input current is discontinuous. Therefore, the circuit is not appropriate for renewable energy applications such as PV applications. The converter efficiency decreased as the number of cells increased because each cell is made up of four components, making it suitable only for low-power applications. Quasi Z-source converters^23^, quasi-Y-source converters^24^, and hybrid Z-source boost converters^25^ were also founded. Two cascaded Z-source boost converters were proposed in^26^, which took the advantage of cascaded technique and impedance source converters. These topologies attained high voltage gain at a small duty ratio and continual input current. But they have a limited duty cycle, excessive stresses on the active switch, and complex drive circuit. A hybrid topology was presented in^27,28^ integrated the coupled inductor and switched capacitor to achieve higher voltage gain. Nonetheless, the copper loss and the main switch voltage stress were raised by rising the turns ratio of the coupled inductor. A DC/DC converter based on quad switched inductor was studied in^29^ to accomplish high voltage gain. However, this converter has a lot of components, which reduces the efficiency, and raises the cost and the size of the circuit. Another DC/DC converter based on the voltage multiplier structure was introduced in^30^. High voltage gain and high efficiency can be achieved, but the converter has some drawbacks for example high voltage stress on the main switch, and is suitable only for low voltage low power applications.

This article offers a new high voltage gain non-isolated DC/DC boost converter to improve the abovementioned problems. This converter uses a reduced number of passive components. It has the following features: control simplicity, high output voltage gain, and small voltage stress across the power switch and diodes. Furthermore, cascading additional cells can be offered to have a very high voltage gain. The circuit description and modes of operation are described in detail. The steady-state process of the proposed converter is studied. The voltage gain derivation in CCM is evaluated. The voltage stresses across the power switch, diodes, and capacitors are derived. Power losses and efficiency are derived based on the equivalent model for the suggested topology circuit with parasitic elements. The experimental results and comparative analysis are given to confirm the success of the proposed converter.

The proposed topology in this paper differs from the circuit in^22^, which uses the main switch connected in series with the input DC source. Consequently, the input current is discontinuous which causes constraints for PV applications. Also, each cell of this circuit in^22^ consists of four components, then, the converter efficiency decreased as the number of cells increases. So, it is suitable only for low-power applications. The current work also differs from the circuits in^23–26^ which employ Quasi Z-source, quasi-Y-source, hybrid Z-source, and two cascaded Z-source boost converters, respectively. Although these topologies attain high voltage gain with a low duty ratio and continuous input current. However, they have a limited duty cycle, high stresses on the active switch, and complex drive circuits.

## The proposed converter and its steady-state investigation

### Circuit description and modes of operation

**Figure 1:**
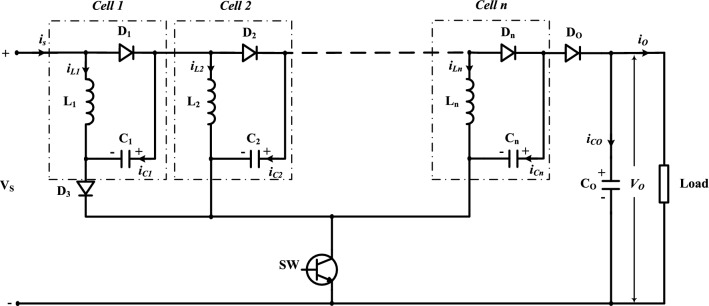
The multi-cells proposed DC-DC boost converter.

**Figure 2:**
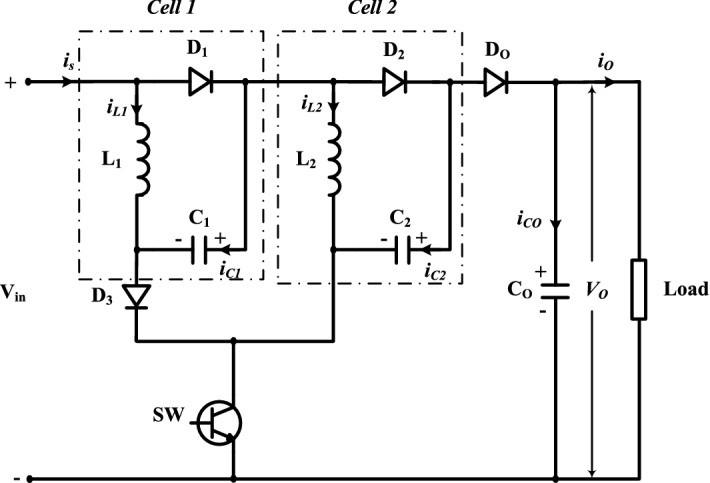
The two cells proposed DC-DC boost converter.

The offered multi-cells DC/DC boost converter is shown in Fig. 1. To understand the operation of the proposed DC-DC boost converter, the circuit with two cells (*n* = 2) shown in Fig. 2 is analyzed. Each cell contains two passive elements (one inductor and one capacitor), and one diode.

**Figure 3:**
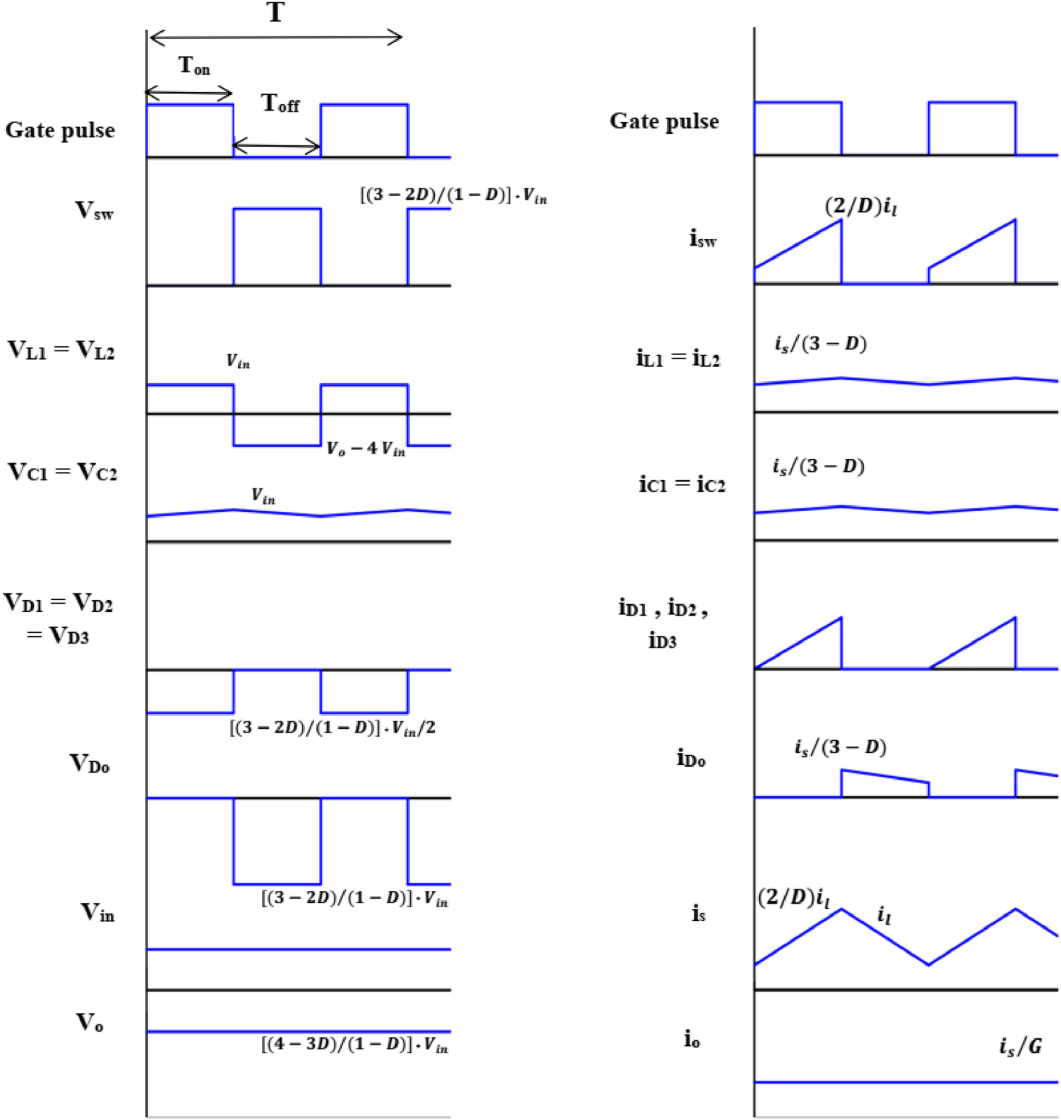
Operating modes of proposed converter.

The proposed circuit composes of one active power switch, four diodes, and five passive components. Under assumptions of the ideal power switch and diodes, and pure inductive and capacitive elements, steady state analysis of the two operation modes is discussed. The key waveforms of the two cells of the suggested converter are displayed in Fig. 3.

#### Mode 1 (0 ≤ t ≤ DT)

**Figure 4:**
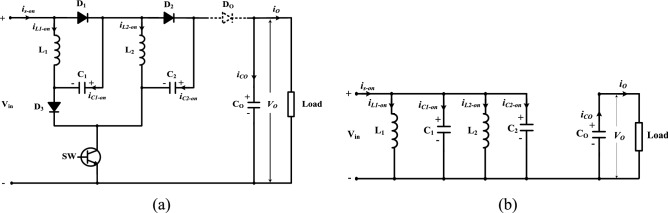
Equivalent circuit of Mode 1: (**a**) active and inactive elements, and (**b**) equivalent electrical circuit.

Figure 4 shows the equivalent circuit during mode 1. As obvious SW, D_1_, D_2_, and D_3_ are ON, and D_o_ is OFF. The currents (*i*_L1_*, i*_L2_) of the inductors (L_1_ and L_2_) increase linearly. The inductor’s voltage opposes the input dc voltage V_*in*_. The capacitors *C*_*1*_ and *C*_*2*_ charge with a voltage nearly the same as V_*in*_. The saved energy in the capacitor *C*_*o*_ supplies the load. The equations of mode 1 can be obtained from Fig. 4b by applying KVL and KCL as follow:

1 $$V_{L1 - on} = V_{L2 - on} = V_{C1 - on} = V_{C2 - on} = V_{in}$$

The supply current during the switching-on period (*i*_s-on_) is given by;

2 $$\left. \begin{gathered} i_{s - on} = i_{L1 - on} + i_{C1 - on} + i_{L2 - on} + i_{C2 - on} \hfill \\ i_{Co} = i_{o} \hfill \\ \end{gathered} \right\}$$

#### Mode 2 (DT ≤ t ≤ T)

**Figure 5:**
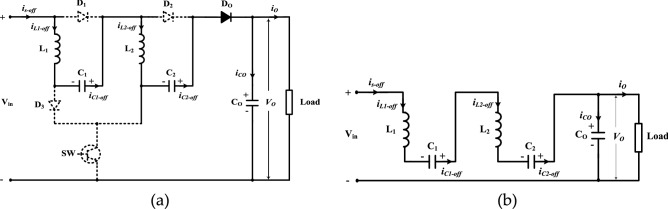
Equivalent circuit of Mode 2: (**a**) active and inactive elements, and (**b**) equivalent electrical circuit.

Figure 5 displays the equivalent circuit during mode 2. It is noted that SW, D_1_, D_2_, and D_3_ are OFF, and D_o_ is ON. The inductors’ voltages V_*L1*_ and V_*L2*_ are inverted, and the saved energy in the passive components *L*_*1*_*, L*_*2*_, *C*_*1,*_ and *C*_*2*_ is transmitted to the load and *C*_*o*_. The equations of mode 2 can be obtained from Fig. 5b by applying KVL and KCL as follow:

3 $$V_{in} + V_{L1 - off} + V_{C1 - off} + V_{L2 - off} + V_{C2 - off} = V_{o}$$

The supply current during the switching-off period (*i*_*s-off*_) is given by;

4 $$\left. \begin{gathered} i_{s - off} = i_{L1 - off} = i_{C1 - off} = i_{L2 - off} = i_{C2 - off} \hfill \\ i_{s - off} = i_{Co} + i_{o} \hfill \\ \end{gathered} \right\}$$

### Voltage gain derivation and voltage stresses across the switch, diodes, and capacitors

The ripple of the inductor current *i*_L1_ during the switching-on interval is:

5 $$\Delta i_{{L_{1 - on} }} = \frac{{V_{in} }}{{L_{1} }}DT$$

where *D* is the duty ratio.

The ripple of the inductor current *i*_L2_ during the switching-on interval is:

6 $$\Delta i_{{L_{2 - on} }} = \frac{{V_{in} }}{{L_{2} }} DT$$

The ripple of the inductor current *i*_L1_ during the switching-off interval is:

7 $$\Delta i_{{L_{1 - off} }} = \frac{{( V_{o} - 4 V_{in} )}}{{L_{1} }} (1 - D) T$$

The ripple of the inductor current *i*_L2_ during this mode is:

8 $$\Delta i_{{L_{2 - off} }} = \frac{{( V_{o} - 4 V_{in} )}}{{L_{2} }} (1 - D) T$$

By using the volt-sec balance through the inductors *L*_1_ and *L*_2_, and from Eqs. (5) and (7);

9 $$\Delta i_{{L_{1} }} = \frac{{V_{in} }}{{L_{1} }} D T = \frac{{( V_{o} - 4 V_{in} )}}{{L_{1} }} (1 - D) T$$

Also, Eqs. (6) and (8) yield;

10 $$\Delta i_{{L_{2} }} = \frac{{V_{in} }}{{L_{2} }} D T = \frac{{( V_{o} - 4 V_{in} )}}{{L_{2} }} (1 - D) T$$

By solving Eqs. (9) or (10), the voltage gain can be found as follow:

11 $$\frac{{V_{o} }}{{V_{in} }} = \frac{{(4 - 3 D)}}{(1 - D)}$$

For steady-state operation, the charge on capacitors *C*_*1*_ and *C*_*2*_ should not change;

12 $$D T i_{C1 - on} = (1 - D) T i_{C1 - off}$$

and

13 $$D T i_{C2 - on} = (1 - D) T i_{C2 - off}$$

If each of the inductances *L*_1_ and *L*_2_ is large enough, *I*_*L*1_ is nearly equal to its average current *I*_*L*1_ and also, *I*_*L*2_ is nearly equal to its average current *I*_*L*2_.

For *L*_1_ = *L*_2_;

14 $$I_{L1} = I_{L2} = I_{L}$$

Then, from Eq. (4), we obtain

15 $$i_{s - off} = I_{L}$$

Also, by substituting from Eqs. (12), (13), and (14) into Eq. (2), one can obtain:

16 $$i_{s - on} = I_{L} + \frac{1 - D}{D}I_{L} + I_{L} + \frac{1 - D}{D}I_{L} = \frac{2}{D}I_{L}$$

The average supply current can be given as:

17 $$I_{s} = D i_{s - on} + (1 - D) i_{s - off}$$

18 $$I_{s} = 2 I_{L} + (1 - D) I_{L} = (3 - D) I_{L}$$

The voltage stresses across the switch, diodes, and capacitors respectively can be expressed as:

19 $$V_{SW} = \frac{{(3 - 2 D)}}{(1 - D)} V_{in}$$

20 $$\left. \begin{gathered} V_{D1} = V_{D2} = V_{D3} = \frac{{(3 - 2 D)}}{{2 (1 - D)}} V_{in} \hfill \\ V_{Do} = \frac{{(3 - 2 D)}}{(1 - D)} V_{in} \hfill \\ \end{gathered} \right\}$$

21 $$\left. \begin{gathered} V_{C1} = V_{C2} = V_{in} \hfill \\ V_{Co} = \frac{{(4 - 3 D)}}{(1 - D)} V_{in} \hfill \\ \end{gathered} \right\}$$

From this analysis, we can summarize the following:

For *n* = 1;

22 $$G_{1} = \frac{(2 - D)}{{(1 - D)}}$$

where *G*_1_ is the voltage gain at number of cells (*n*) = 1.

For *n* = 2;

23 $$G_{2} = \frac{{(4 - 3 D)}}{(1 - D)}$$

This equation can be written as;

24 $$G_{2} = \frac{{(2 - D)}}{(1 - D)} + 2$$

where *G*_2_ is the voltage gain at number of cells (*n*) = 2.

For *n* = 3;

25 $$G_{3} = \frac{{(6 - 5 D)}}{(1 - D)}$$

This equation can be written as:

26 $$G_{3} = \frac{{(2 - D)}}{(1 - D)} + 4$$

where *G*_3_ is the voltage gain at number of cells (*n*) = 3.

To generalize the formula based on number of cells (*n*), the voltage gain can be obtained according to the following formula for *n* ≥ 1.

27 $$G_{n} = \frac{{(2 - D)}}{(1 - D)} + 2(n - 1)$$

and the average supply current for any number of cells can be given as following:

28 $$I_{s} = ( n + 1 - D) I_{L}$$

**Figure 6:**
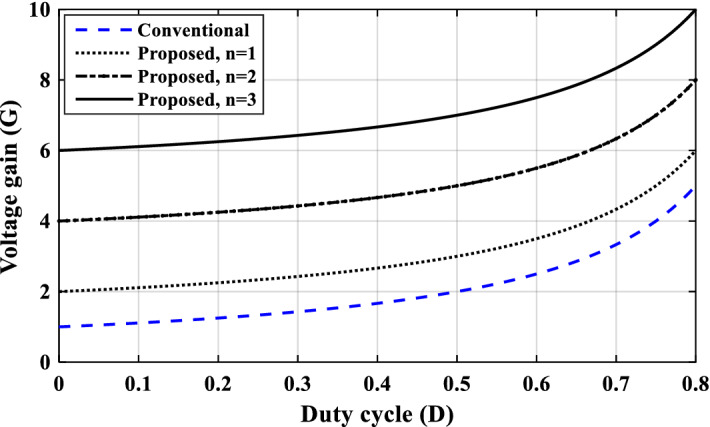
Voltage gain of the proposed boost converter at different number of cells (*n* = 1, 2 and 3) and the conventional one.

The voltage gain of the proposed converter at different number of cells (*n* = 1, 2, and 3) is displayed in Fig. 6. It is obvious that the voltage gain has a higher value in comparison to the conventional DC-DC boost converter. Moreover, the proposed converter can be extended to get higher voltage gains by increasing the cascading additional cells.

Also, the voltage stresses across the switch, diodes, and capacitors for any number of cells can be expressed as:

29 $$V_{SW} = (G_{n} - 1) V_{in}$$

30 $$\left. \begin{gathered} V_{D1} = V_{D2} = V_{D3} = \frac{{(G_{n} - 1)}}{2} V_{in} \hfill \\ V_{Do} = (G_{n} - 1) V_{in} \hfill \\ \end{gathered} \right\}$$

31 $$\left. \begin{gathered} V_{C1} = V_{C2} = V_{in} \hfill \\ V_{Co} = G_{n} V_{in} \hfill \\ \end{gathered} \right\}$$

### Design consideration

The design of inductors and capacitors is considered an important issue to guarantee the operation of the suggested circuit in CCM. Therefore, this part is presented to describe the design of these passive elements.

#### Inductor design

During Mode 1, the voltage across the inductor is provided by:

32 $$V_{L} = L\frac{di}{{dt}} = L\frac{{\Delta I_{L} }}{{\Delta t_{on} }}$$

The voltage across the inductor is the same as the input voltage. The inductance is defined by:

33 $$L = \frac{{V_{in} }}{{\Delta I_{L} }} D T = \frac{{V_{in} }}{{{(\% }r_{i} ) I_{L} }} D T$$

where %*r*_i_ is the percent inductor current ripple allowed. The average value of the inductor current *I*_*L*_ can be obtained from Eq. (18), then, substituting into Eq. (33), one obtains:

34 $$L = \frac{{D (3 - D)}}{{{(\% }r_{i} ) G_{2}^{2} }} R_{L} T$$

where *R*_*L*_ is the load value. Then, the inductance value for any number of cells can be given from the following equation,

35 $$L = \frac{{D (n + 1 - D)}}{{{(\% }r_{i} ) G_{n}^{2} }} R_{L} T$$

#### Capacitor design

The current flowing through the capacitor *C*_*1*_ in *0* ≤ *t* ≤ *DT* period can be obtained from Eqs. (12) and (15) as ((1-*D*)**/***D*)*I*_*L*_. Then, the capacitance value *C*_*1*_ can be derived as:

36 $$C_{1} \frac{{\Delta V_{C} }}{{D T}} = C_{1} \frac{{{(\% }r_{v} ) V_{in} }}{{D T}} = \frac{(1 - D)}{D} I_{L}$$

where %*r*_v_ is the percent capacitor voltage ripple allowed in *C*_*1*_.

By substituting from Eq. (18) into Eq. (36), the capacitance value *C*_*1*_ can be obtained. In the same way, the capacitance value *C*_*2*_ can be derived. Hence,

37 $$C_{1} = C_{2} = \frac{{G_{2}^{2} T}}{{{(\% }r_{v} ) R_{L} }} * \frac{(1 - D)}{{(3 - D)}}$$

For any number of cells, the capacitance value of *C*_*1*_ and *C*_*2*_ can be given from the following relation:

38 $$C_{1} = C_{2} = \frac{{G_{n}^{2} T}}{{{(\% }r_{v} ) R_{L} }} * \frac{(1 - D)}{{(n + 1 - D)}}$$

The capacitor *C*_*o*_ is charged in the period *DT* ≤ *t* ≤ *T*, hence,

39 $$C_{o} = \frac{{(1 - D) T}}{{\Delta V_{Co} }}* I_{{C_{o} }} = \frac{{(1 - D) T}}{{{(\% }r_{v} ) V_{Co} }}*(I_{L} - I_{o} )$$

By substituting from Eq. (18) into Eq. (39), the capacitance value *C*_*o*_ can be obtained.

40 $$C_{o} = \frac{{(1 - D) T}}{{{(\% }r_{v} ) R_{L} }}*(\frac{{G_{2} }}{(3 - D)} - 1)$$

For any number of cells, the capacitance value of *C*_*o*_ can be given from the following relation,

41 $$C_{o} = \frac{{(1 - D) T}}{{{(\% }r_{v} ) R_{L} }}*(\frac{{G_{n} }}{(n + 1 - D)} - 1)$$

## Power losses and efficiency

**Figure 7:**
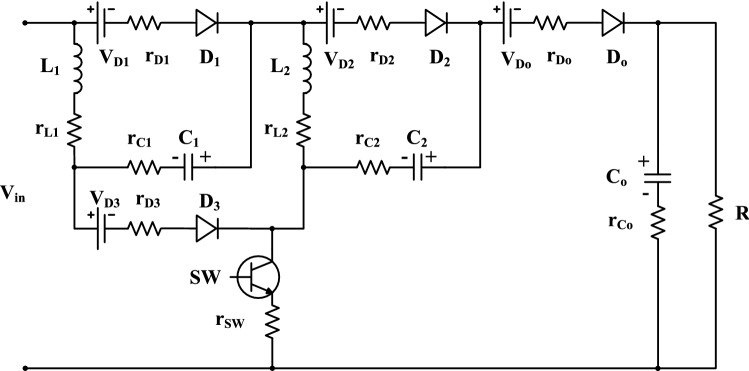
Equivalent model for the proposed circuit with parasitic elements.

The power losses of the two-cell for the proposed topology are estimated by calculating the switching losses and conduction losses. The power loss of each component is determined, then, the converter power losses can be investigated by summing all these parts. Also, the converter efficiency can be determined based on the power losses. The converter model with the parasitic elements is displayed in Fig. 7.

For the computation of conduction losses in the converter, all diodes are considered with cut in voltages *V*_*D1*_, *V*_*D2*_, *V*_*D3*_, and *V*_*Do*_. Also, the internal resistances are *r*_*D1*_, *r*_*D2*_, *r*_*D3*_, and *r*_*Do*_. Each inductor *L*_*1*_ and *L*_*2*_ has a lumped DC resistance *r*_*L1*_ and *r*_*L2*_, respectively, and each capacitor *C*_*1*_, *C*_*2*_ and *C*_*o*_ has an equivalent series resistance *r*_*C1*_, *r*_*C2*_, and *r*_*Co*_, respectively. Both conduction and switching losses are considered for the main switch with on-state resistance occupied as *r*_*sw*_.

### The power switch losses

The practical power switch has conduction and switching losses. The switching loss is the sum of the conduction and switching losses and it can be written as:

42 $$P_{{loss (SW)}} = P_{{loss - conduction (SW)}} + P_{{loss - switching (SW)}}$$

where the conduction loss of SW can be stated as:

43 $$P_{{loss - conduction (SW)}} = i_{SWrms}^{2} *r_{SW}$$

The switch current can be obtained from Mode 1 and Eq. (16), then the *rms* value of the switch current can be established as:

44 $$i_{SWrms} = \frac{{2 V_{o} G}}{{R \sqrt D (3 - D)}}$$

Substituting from (44) into (43), then the power conduction loss can be determined by:

45 $$P_{{loss - conduction (SW)}} = \frac{{4 V_{o}^{2} G^{2} }}{{R^{2} D (3 - D)^{2} }}r_{SW}$$

The switching loss (*P*_loss-switching_) of the power switch SW can be determined by:

46 $$\begin{aligned} P_{{loss - switching (SW)}} & = P_{{loss - switching (SW) - on}} + P_{{loss - switching (SW) - off}} \\ P_{{loss - switching (SW)}} & = \frac{{t_{on} *V_{SW} *i_{SW - on} *f_{S} }}{2} + \frac{{t_{off} *V_{SW} *i_{SW - off} *f_{S} }}{2} \\ P_{{loss - switching (SW)}} & = \frac{{(t_{rt} + t_{ft} )*V_{SW} *i_{SW - avg} *f_{S} }}{2} \\ \end{aligned}$$

where *t*_*rt*_ and *t*_*ft*_ is the rise time and fall time of the switch, respectively.

The average current of the switch current can be found as:

47 $$i_{SW - avg} = \frac{{2 G V_{o} }}{{R (3 - D)}}$$

Substituting from (19) and (47) into (46), then the power switching loss can be determined by:

48 $$P_{{loss - switching (SW)}} = \frac{{(t_{rt} + t_{ft} )*V_{o}^{2} *(3 - 2D)*f_{S} }}{{R (3 - D) (1 - D)}}$$

Then the switching loss of SW can be investigated by:

49 $$\left. \begin{aligned} P_{{loss - total (SW)}} & = \frac{{4 G^{2} V_{o}^{2} }}{{R^{2} D (3 - D)^{2} }}r_{SW} \\ & \quad + \frac{{(t_{rt} + t_{ft} )*V_{o}^{2} *(3 - 2D)*f_{S} }}{{R (3 - D) (1 - D)}} \\ \end{aligned} \right\}$$

### The diodes losses

The diodes are supposed to have the same cut in voltages and equivalent series resistance, *V*_*D1*_ = *V*_D2_ = *V*_D3_ = *V*_Do_ = *V*_D ;_ *r*_D1 =_ *r*_D2 =_ *r*_D3 =_ *r*_Do =_ *r*_D_.

The total diodes losses can be stated as:

50 $$\left. \begin{aligned} P_{{loss - total (Diodes)}} &= P_{loss - D1} + P_{loss - D2} \hfill \\ &\quad + P_{loss - D3} + P_{loss - Do} \hfill \end{aligned} \right\}$$

where, the power loss of each diode can be determined by;

51 $$\begin{aligned} P_{loss - D1} & = V_{D} *i_{D1avg} + i_{D1rms}^{2} *r_{D} \\ P_{loss - D1} & = (2 - D) V_{D} *i_{L} + \frac{{(2 - D)^{2} }}{D}*i_{L}^{2} *r_{D} \\ P_{loss - D1} & = \frac{{(2 - D) G V_{o} V_{D} }}{{R (3 - D)}} + \frac{{(2 - D)^{2} G^{2} V_{o}^{2} }}{{R^{2} (3 - D)^{2} D}}*r_{D} \\ \end{aligned}$$

52 $$\begin{aligned} P_{loss - D2} & = V_{D} *i_{D2avg} + i_{D2rms}^{2} *r_{D} \\ P_{loss - D2} & = (1 - D) V_{D} *i_{L} + \frac{{(1 - D)^{2} }}{D}*i_{L}^{2} *r_{D} \\ P_{loss - D2} & = \frac{{(1 - D) G V_{o} V_{D} }}{{R (3 - D)}} + \frac{{(1 - D)^{2} G^{2} V_{o}^{2} }}{{R^{2} (3 - D)^{2} D}}*r_{D} \\ \end{aligned}$$

53 $$\begin{aligned} P_{loss - D3} & = V_{D} *i_{D3avg} + i_{D3rms}^{2} *r_{D} \\ P_{loss - D3} & = V_{D} *i_{L} + \frac{1}{D}*i_{L}^{2} *r_{D} \\ P_{loss - D3} & = \frac{{G V_{o} V_{D} }}{{R (3 - D)}} + \frac{{G^{2} V_{o}^{2} }}{{R^{2} (3 - D)^{2} D}}*r_{D} \\ \end{aligned}$$

54 $$\begin{aligned} P_{loss - Do} & = V_{D} *i_{Doavg} + i_{Dorms}^{2} *r_{D} \\ P_{loss - Do} & = (1 - D) V_{D} *i_{L} + (1 - D)*i_{L}^{2} *r_{D} \\ P_{loss - Do} & = \frac{{(1 - D) G V_{o} V_{D} }}{{R (3 - D)}} + \frac{{(1 - D) G^{2} V_{o}^{2} }}{{R^{2} (3 - D)^{2} }}*r_{D} \\ \end{aligned}$$

Then, the total power loss in the diodes can be determined by substituting from (51), (52), (53), and (54) into (50) as:

55 $$\left. \begin{aligned} P_{{loss - total (Diodes)}} &= \frac{{(5 - 3D) G V_{o} V_{D} }}{{R (3 - D)}} \hfill \\ &\quad + \frac{{(2 - D) G^{2} V_{o}^{2} }}{{R^{2} (3 - D) D}}*r_{D} \hfill \\ \end{aligned} \right\}$$

### The capacitors losses

There are three capacitors are shown in the proposed topology. The total power loss due to the capacitors is given by:

56 $$\left. \begin{aligned} P_{{loss - total (Capacitors)}} &= i_{{_{C1rms} }}^{2} *r_{C1} + i_{{_{C2rms} }}^{2} *r_{C2} \hfill \\ &\quad+ i_{{_{Corms} }}^{2} *r_{Co} \hfill \end{aligned} \right\}$$

The three capacitors are assumed to has the same equivalent series resistance, *r*_*C1*_ = *r*_*C2*_ = *r*_*Co*_ = *r*_*C*_*.*

The *rms* value of current through the capacitors and the power loss of each capacitor can be estimated using the expressions:

57 $$\left. \begin{gathered} i_{C1rms} = i_{C2rms} = \sqrt {\frac{(1 - D)}{D}} I_{L} \hfill \\ i_{Corms} = \sqrt {\frac{D}{(1 - D)}} I_{o} \hfill \\ \end{gathered} \right\}$$

Then,

58 $$\left. \begin{gathered} P_{loss - C1} = P_{loss - C2} = \frac{(1 - D)}{D} I_{L}^{2} *r_{C} = \frac{(1 - D)}{D} \frac{{G^{2} V_{o}^{2} }}{{R^{2} (3 - D)^{2} }}*r_{C} \hfill \\ P_{loss - Co} = \frac{D}{(1 - D)} I_{o}^{2} *r_{C} = \frac{{D V_{o}^{2} }}{{R^{2} (1 - D)}} *r_{C} \hfill \\ \end{gathered} \right\}$$

Substituting from (58) into (56), the total power loss due to the capacitors can be given by:

59 $$\left. \begin{aligned} P_{{loss - total (Capacitors)}} &= \frac{{2 (1 - D)}}{D} \frac{{G^{2} V_{o}^{2} }}{{R^{2} (3 - D)^{2} }}*r_{C} \hfill \\ &\quad + \frac{{D V_{o}^{2} }}{{R^{2} (1 - D)}} *r_{C} \hfill \end{aligned} \right\}$$

### The inductors losses

The inductors loss can be expressed as:

$$P_{{loss - total (Inductors)}} = i_{{_{L1rms} }}^{2} *r_{L1} + i_{{_{L2rms} }}^{2} *r_{L2}$$

Assuming the inductors have the same internal resistance *r*_*L1*_ = *r*_*L2*_ = *r*_*L*_ and have the same *rms* value of the inductor currents *i*_*L1rms*_ = *i*_*L2rms*_ = *i*_*Lrms*_. Then, the inductors losses can be determined as:

60 $$P_{{loss - total (Inductors)}} = 2 i_{{_{Lrms} }}^{2} r_{L}$$

Using (18), the *rms* value of the inductor current can be established, then the total power loss in the inductors can be expressed as;

61 $$P_{{loss - total (Inductors)}} = \frac{{2 G^{2} V_{o}^{2} }}{{R^{2} (3 - D)^{2} }}*r_{L}$$

Substituting from Eqs. (49), (55), (59), and (61) into the below equation, the total converter loss can be obtained.

62 $$\left. \begin{aligned} P_{loss - total} &= P_{{loss - total (Switches)}} + P_{{loss - total (Diodes)}} \hfill \\ &\quad + P_{{loss - total (Capacitors)}} + P_{{loss - total (Inductors)}} \hfill \end{aligned} \right\}$$

The expression for total losses is as follows:

63 $$\left. \begin{aligned} P_{loss - total} &= [a ( 4 \frac{{r_{SW} }}{R} + b \frac{{r_{D} }}{R}) + c \frac{{r_{C} }}{R} + d \frac{{r_{L} }}{R} \hfill \\ &\quad + e \frac{{V_{D} }}{{V_{o} }} + f((t_{rt} + t_{ft} ) f_{s} ) ] P_{o} \hfill \\ \end{aligned} \right\}$$

where

64 $$\left. \begin{gathered} a = \frac{{G^{2} }}{{D (3 - D)^{2} }}, b = (2 - D)(3 - D) \hfill \\ c = \frac{{2 G^{2} (1 - D)}}{{D (3 - D)^{2} }} + \frac{D}{(1 - D)}, d = \frac{{2 G^{2} }}{{(3 - D)^{2} }} \hfill \\ e = \frac{{(5 - 3D) G}}{(3 - D)}, f = \frac{(3 - 2D)}{{(3 - D) (1 - D)}} \hfill \\ \end{gathered} \right\}$$

Finally, the efficiency (*ɳ*) of the suggested topology can be determined as:

65 $$\eta = \frac{{P_{o} }}{{P_{o} + P_{loss - total} }}$$

where, *P*_*o*_ is the output power. Substituting from (63) into (65), the proposed converter efficiency (*ɳ*) can be defined as:

66 $$\eta = \frac{1}{{1 + a \left( {4 \frac{{r_{SW} }}{R} + b \frac{{r_{D} }}{R}} \right) + c \frac{{r_{C} }}{R} + d \frac{{r_{L} }}{R} + e \frac{{V_{D} }}{{V_{o} }} + f (t_{rt} + t_{ft} ) f_{S} }}$$

## Experimental results

**Figure 8:**
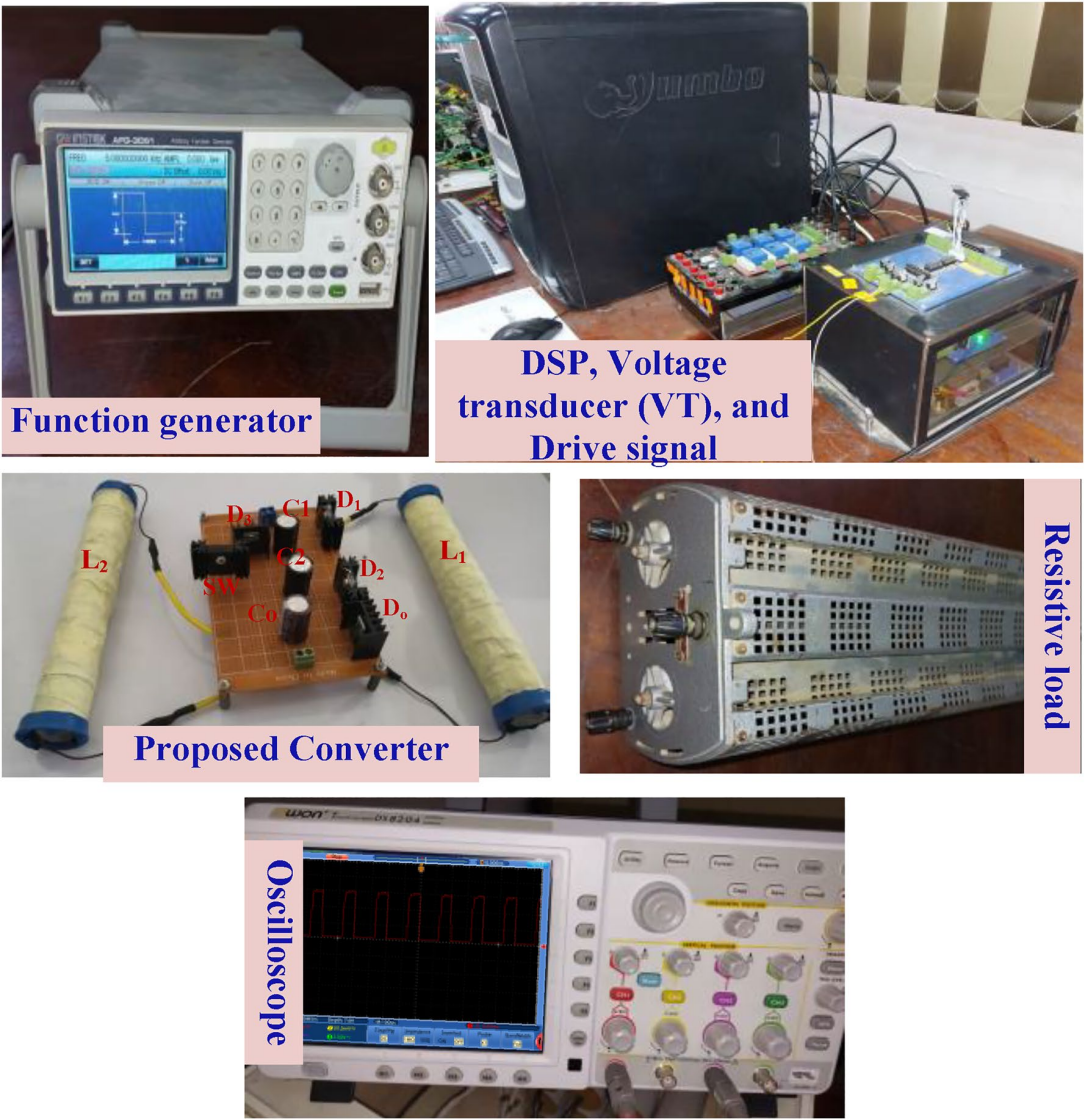
The experimental setup system.

**Figure 9:**
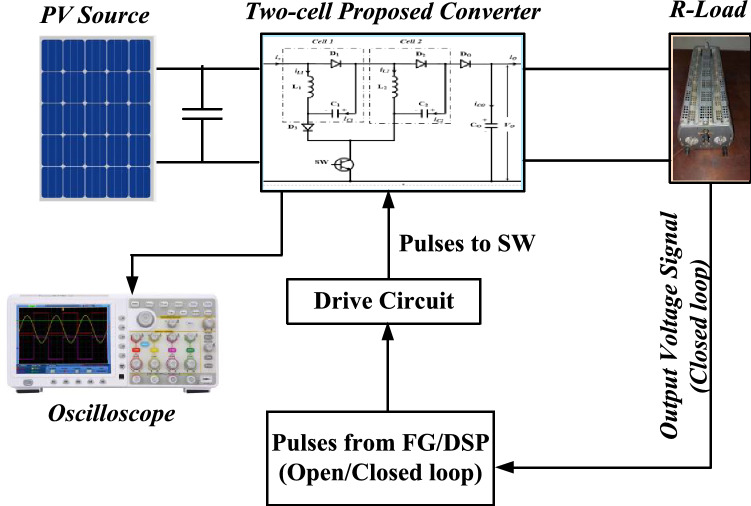
The schematic drawing for the test system.

To authorize the efficacy of the proposed DC-DC boost converter, a prototype has been built in the laboratory. The parameters of the suggested converter are given in Table 1. A photograph of the experimental setup system is shown in Fig. 8, and a schematic drawing for the test system is shown in Fig. 9. The results are captured using an oscilloscope during open-loop and closed-loop control.

**Table 1:** Parameters of the suggested DC–DC boost converter.

Devices	Value
Inductances (L_1_, L_2_, L_3_, L_4_)	9.3 mH
Input voltage (*V*_in_)	24 V
Output voltage (*V*_o_)	107 V
Output power (*P*_o_)	52 W
Load (*R*)	220 Ω
Inductances (*L*_*1*_ and *L*_*2*_)	3 mH
Capacitances (*C*_*1*_, *C*_*2*_)	4.7 μF
Diodes (*D*_*1*_, *D*_*2*_, *D*_*3*_, and *D*_*o*_)	MUR1560
Main switch (SW)	FGH40N60UFD
Switching frequency (*f*_S_)	5 kHz

### Open loop results

**Figure 10:**
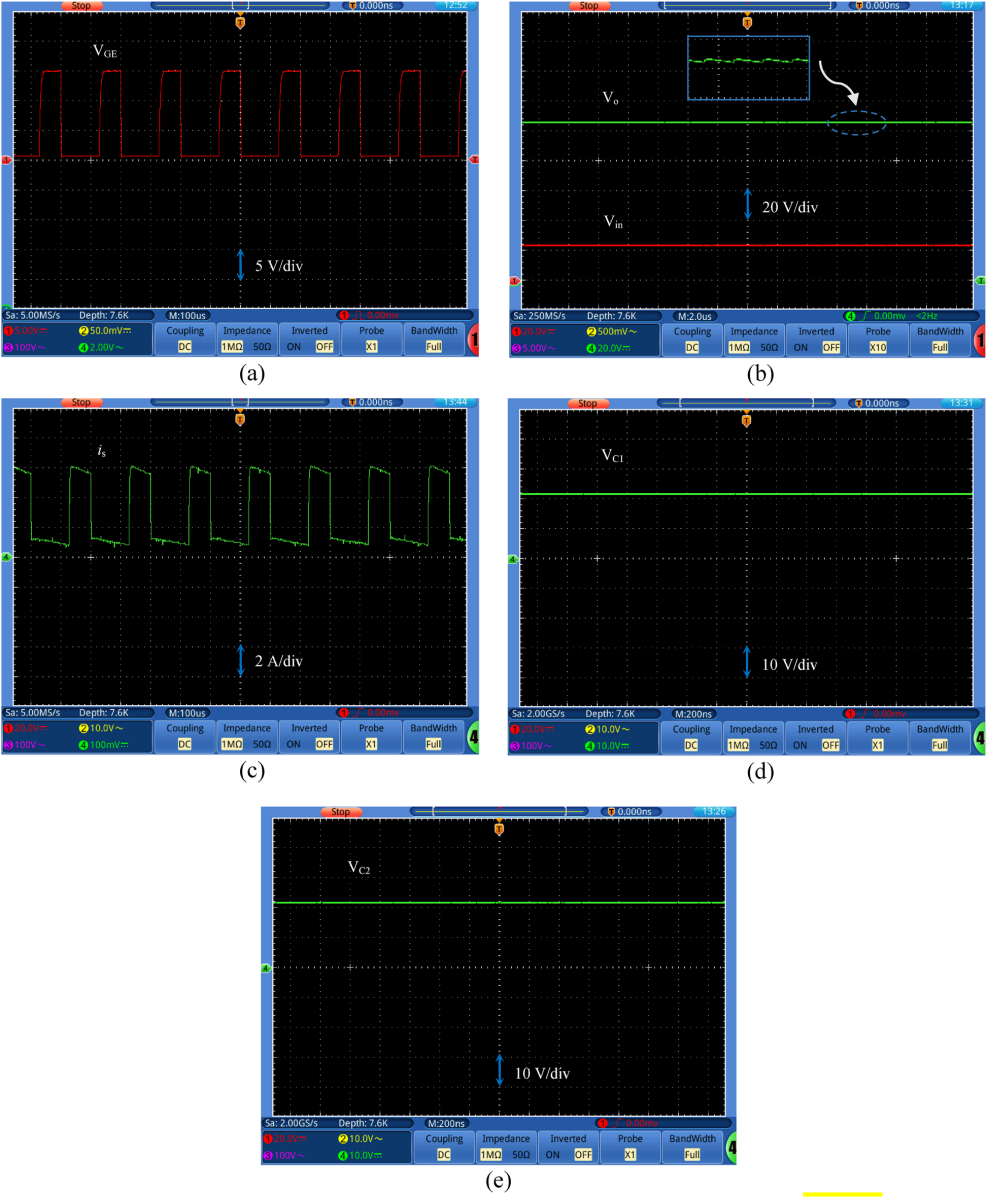
Experimental waveforms of (**a**) gate-emitter voltage, (**b**) input and output voltage, (**c**) input current, (**d**) capacitor *C*_1_ voltage, and (**e**) capacitor *C*_2_ voltage.

**Figure 11:**
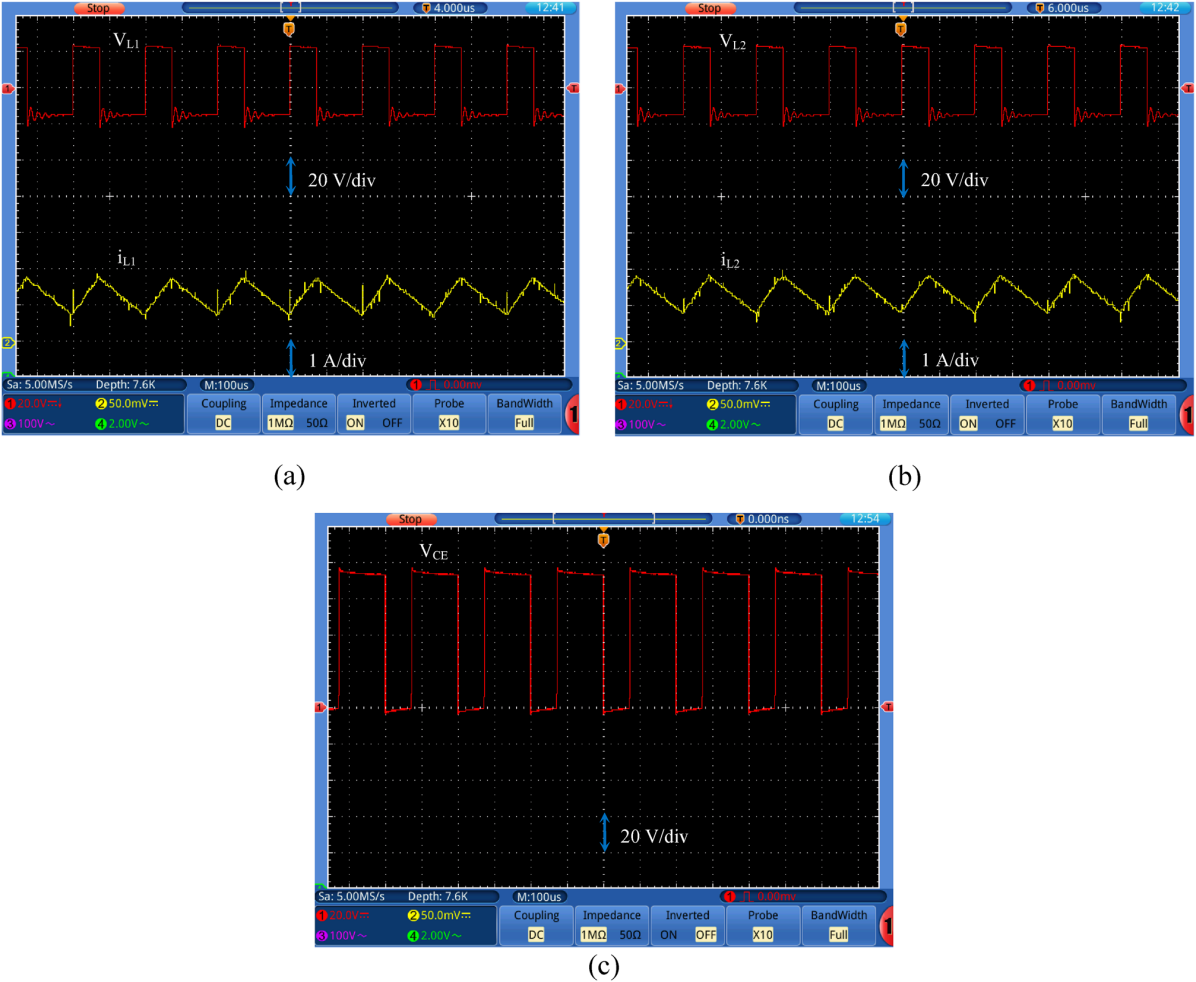
Experimental waveforms of (**a**) inductor *L*_*1*_ voltage and current, (**b**) inductor *L*_*2*_ voltage and current, and (**c**) SW voltage.

**Figure 12:**
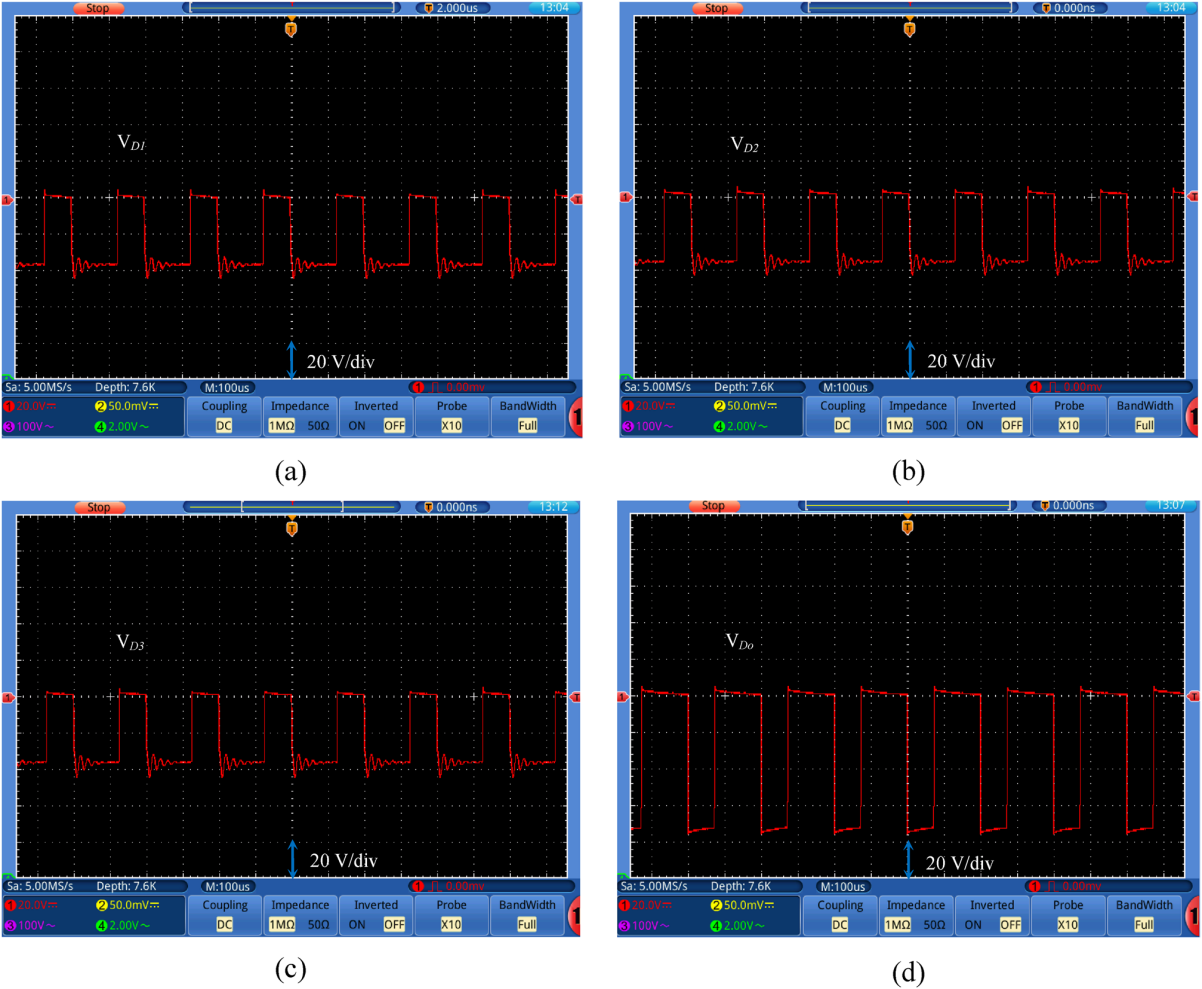
Experimental waveforms of (**a**) diode *D*_*1*_ voltage, (**b**) diode *D*_*2*_ voltage, (**c**) diode *D*_*3*_ voltage, and (**d**) diode *D*_*o*_ voltage.

**Figure 13:**
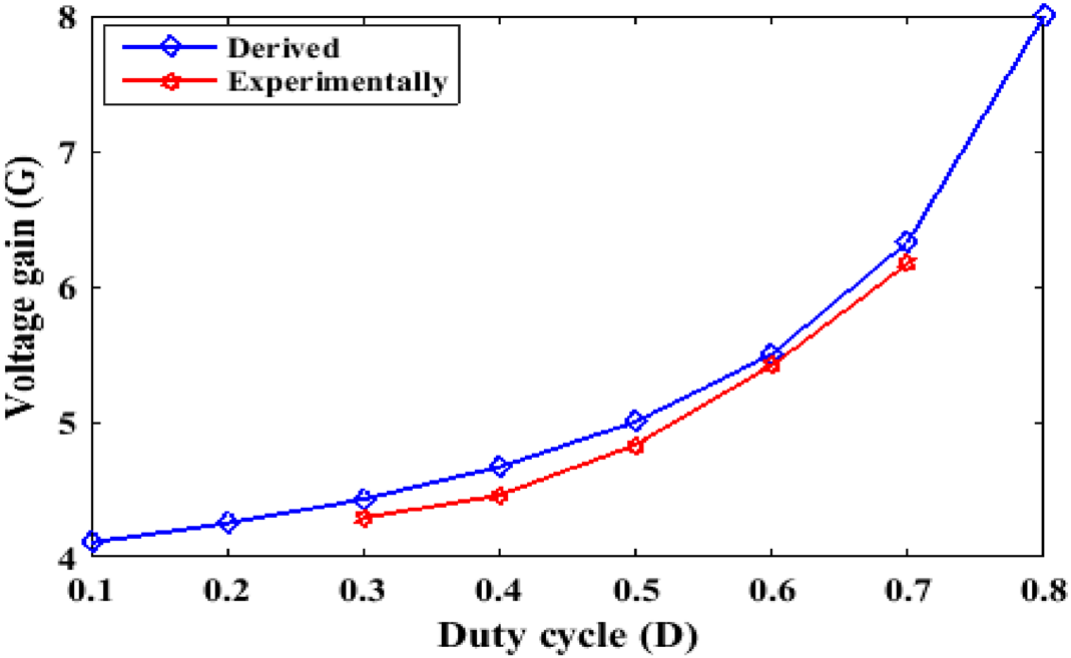
Voltage gain versus duty cycle: derived and experimentally.

Figures 10, 11, 12 and 13 show the experimental results when the suggested DC-DC boost topology operates at *D* = 0.4. Fig. 10a shows the gate-emitter switch voltage waveform with duty cycle 40%. Fig. 10b shows the input and output voltage waveforms of the proposed converter. It is observed that the output DC voltage at this duty cycle equal to about 107 V obtained from input voltage equal to 24 V, that represents 4.46 times of the input voltage. Also, the output voltage ripple is equal to 4 V that represents 3.7% from the output voltage.

It can be noticed from Fig. 10c that the input current is continuous with average value equal to about 2.36 A. The voltages of the capacitors *C*_*1*_ and *C*_*2*_ are shown in Fig. 10d and e, respectively. The voltages across the capacitors *C*_*1*_ and *C*_*2*_ are equal with value 21 V across each capacitor, which validates Eq. (21). The experimental waveforms of voltages and currents for *L*_*1*_ and *L*_*2*_ are captured in Fig. 11a and b, respectively. It can be noticed that i_L1_ and i_L2_ are continuous signals. This ensures that the converter operates in CCM. The average current value passes through L_1_ or L_2_ is found to be 1 A that confirms Eq. (18). The voltage across L_1_ or L_2_ is almost equal to the input voltage (24 V) during the mode 1, and their values are—14 V during mode 2 that confirm the analysis. Figure 11c displays the voltage across the main switch (SW) with a value of 76 V.

The experimental waveforms of voltages across *D*_*1*_, *D*_*2*_, *D*_*3*_, and *D*_*o*_ are shown in Fig. 12 with values of 36.1 V, 35.9 V, 36 V and 76 V, respectively. It can be seen that the voltage across the diodes *D*_*1*_, *D*_*2*_, and *D*_*3*_ is almost equal with a value equal to the half value of the voltage across the diode *D*_*o*_ that confirms Eq. (20). Furthermore, Eq. (19) is validated from Figs. 11c and 12d. These figures show that the voltage through the switch equals to the diode *D*_*o*_ voltage.

To present the voltage gain against the duty cycle, the waveforms of Fig. 13 are plotted for derived and experimental results. It can be observed that the experimental and the theoretical curves have a good convergence. However, the slight variations between the theoretical and experimental results are due to the influence of parasitic elements.

**Figure 14:**
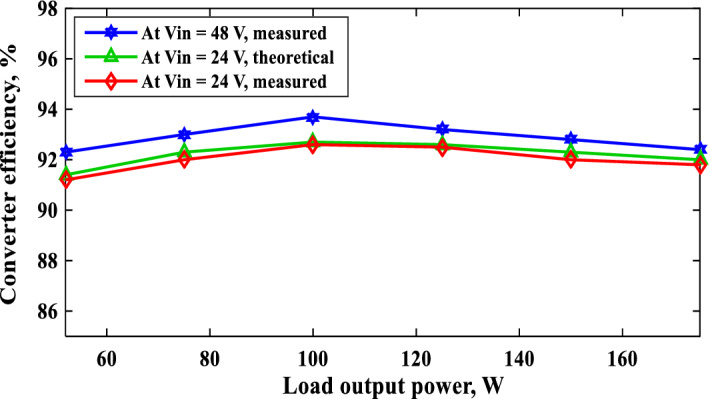
Converter efficiency versus output power at different values of the input voltage and *D* = 0.4.

The converter efficiency against the output power at different values of the input voltage and at *D* = 0.4 is given in Fig. 14. The maximum measured efficiency at *V*_*in*_ = 24 V and *D* = 0.4 is 92.6% and increases to 93.7% if the input voltage increases to 48 V. Also, it can be seen that the efficiency is enhanced with raising the input voltage. Furthermore, the measured efficiency at *V*_*in*_ = 24 V and output power *P*_*o*_ = 52 W is 91.2%. In this case, the total power losses equal 5 W that represents 9.6% from the output power. Also, Fig. 14 shows the theoretical efficiency curve at *V*_*in*_ = 24 V that calculated from Eq. (66). The great convergence between the theoretical and measured efficiency curves proves the validity of the analysis. Table 2 shows the measured efficiency of the suggested converter and other similar works at the same output power, *P*_*o*_ = 52 W. It is clear from Table 2 that the suggested converter has the highest efficiency compared to the other related topologies.

**Table 2:** Measured efficiency of the proposed circuit and other related topologies.

Type of converter	Measured efficiency (%)
Conventional converter	87.3
A converter in^3^	90.8
A converter in^15^	90
A converter in^22^	88.2
A converter in^23^	88
A converter in^24^	83.5
Proposed converter	91.2

**Figure 15:**
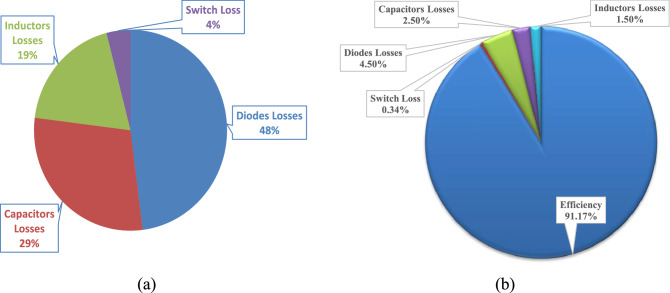
(**a**) Power loss distribution, and (**b**) the efficiency pie chart at a duty cycle of 40%, *V*_*in*_ = 24 V and *P*_*o*_= 52 W for converter components.

Figure 15a illustrates the power losses division and the ratio of power losses for each component from the total power losses. The switch loss, the inductors losses, the capacitors losses, and the diodes losses represents 4%, 19%, 29%, and 48% of the total power losses, respectively. Figure 15b illustrates the efficiency pie chart at this experimental case. Hence, the converter efficiency can be enhanced by choosing low voltage/current rating of its components and the production of final converter fabrication.

### Closed loop results

**Figure 16:**
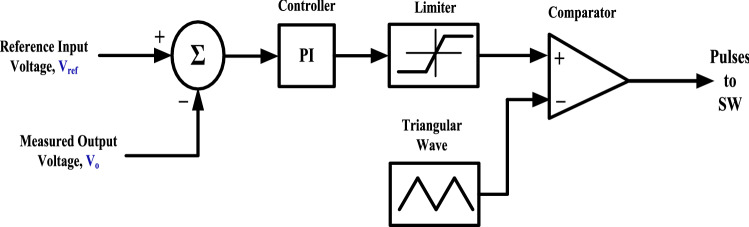
A schematic diagram of closed loop control circuit.

The experimental prototype is also examined during a closed loop control. A schematic diagram of the circuit that is utilized to control the output voltage of the proposed converter is shown in Fig. 16. A PI controller is applied to regulate the output voltage. The parameters of the PI controller are *K*_*P*_ = 100 and *K*_*I*_ = 200.

**Figure 17:**
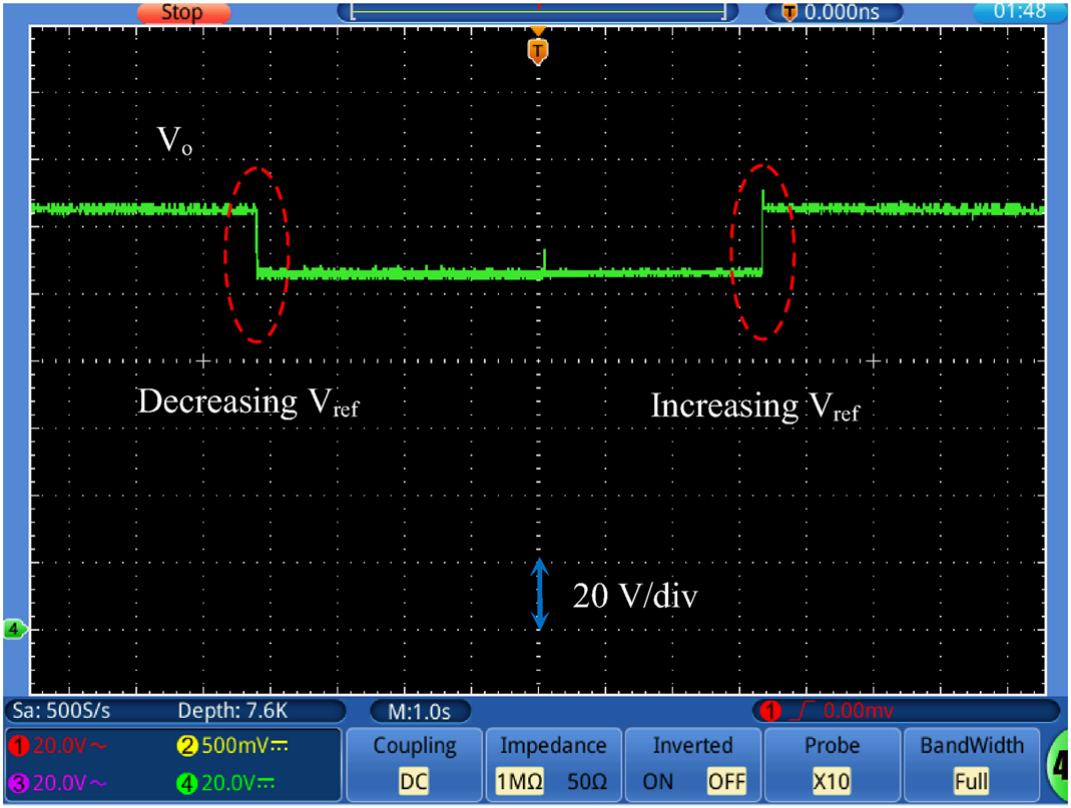
Output voltage with changing the reference voltage (*V*_*ref*_) at *V*_*in*_ = 24 V.

**Figure 18:**
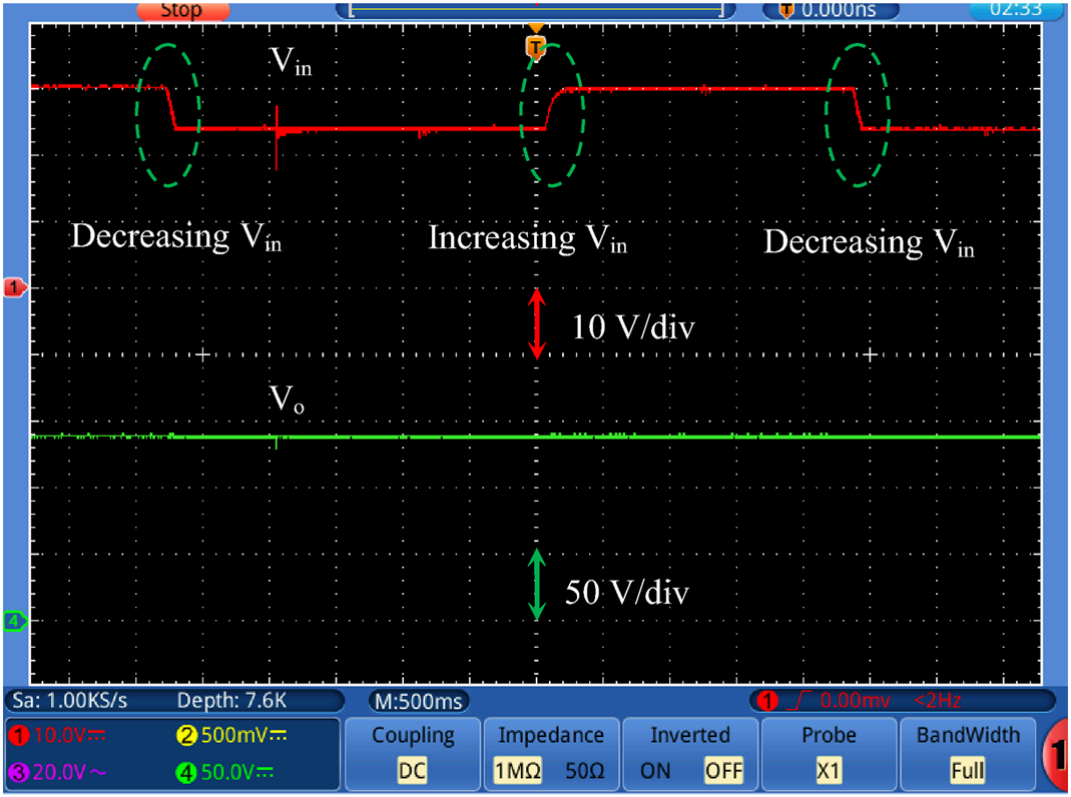
Output voltage response with changing the input voltage (*V*_*in*_) at *V*_*ref*_ = 140 V.

**Figure 19:**
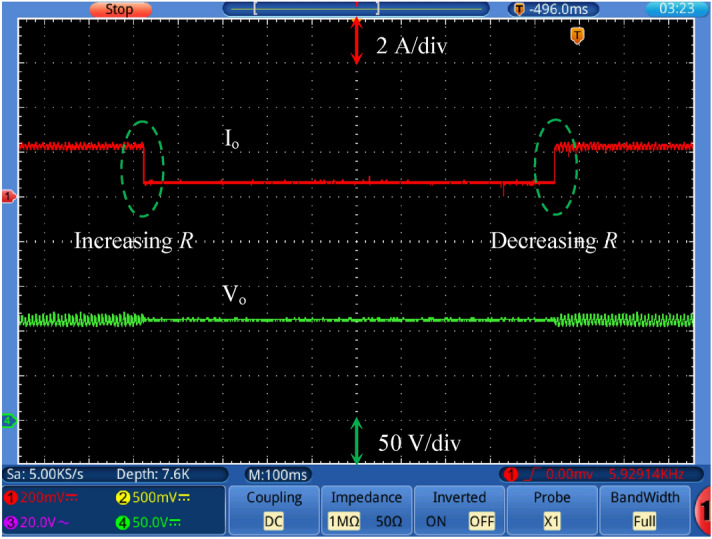
Output voltage response with changing the load value at *V*_*ref*_ = 115 V.

Figure 17 shows the output voltage response with changing the reference voltage (*V*_*ref*_) at input dc voltage equal to 24 V and a full load value. A step change in the reference voltage from 125 to 105 V (decrease) and from 105 to 125 V again (increase). It is noted that the output voltage tracks the reference voltage smoothly. Figure 18 shows the output voltage response with changing the input voltage at *V*_*ref*_ = 140 V. A step change (decrease/increase/decrease) in the input voltage from 30 to 24 V is applied. This step change represents 25% change of the input voltage. It is observed that the output voltage maintains its value under reference voltage change. Figure 19 shows the output voltage response with changing the load resistance value at *V*_*ref*_ = 115 V. A step change (increase/decrease) in the load resistance value from about 30% of the full load to full load value is applied. The output voltage remains constant under load changes. This proves the effectiveness of the closed loop control of the proposed converter.

## Discussion

**Figure 20:**
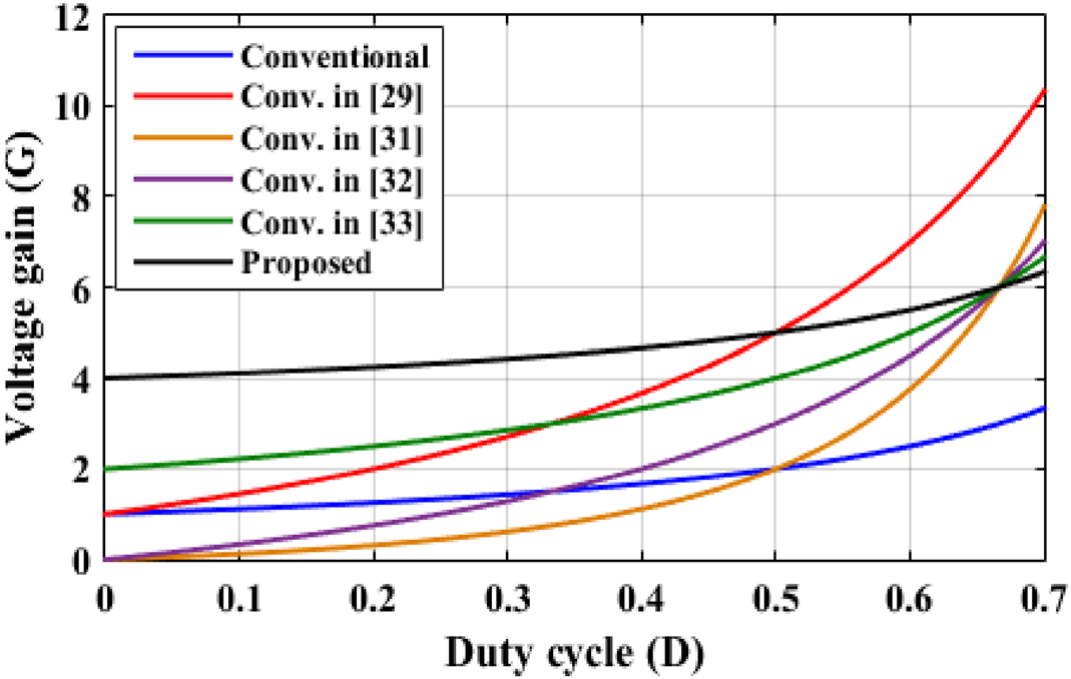
Voltage gain versus duty cycle.

**Figure 21:**
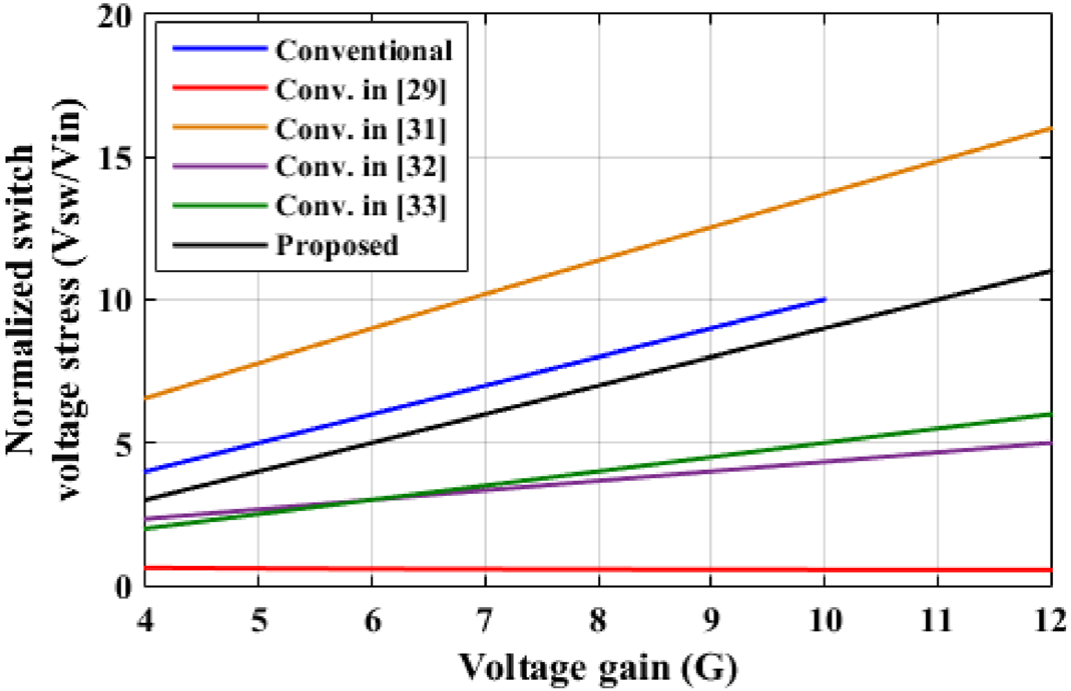
Normalized switch voltage stress with voltage gain variation.

**Figure 22:**
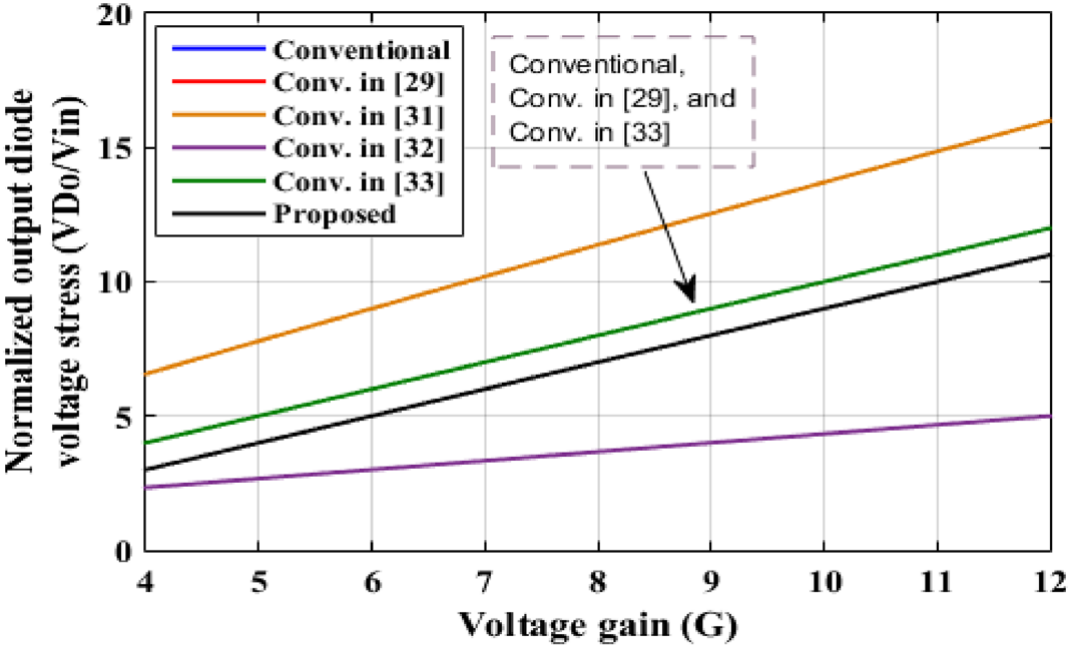
Normalized output diode voltage stress with voltage gain variation.

In this part, a comparison between the two-cell proposed converter and other recent boost topologies is presented. Table 3 shows the comparison results according to the number of components, ideal voltage gain, and maximum voltage stresses through the main switch, output diode, and the output capacitors. The waveforms, which summarize the comparison, are plotted as shown in Figs. 20, 21 and 22.

**Table 3:** Comparison between the proposed converter and recent converter topologies.

Converter	Number of								
	Switches	Inductors	Diodes	Capacitors	All components	Voltage gain (*G*)	Maximum switch voltage stress	Output diode voltage	Output capacitor voltage
Conventional	1	1	1	1	4	$\frac{1}{(1 - D)}$	*GV*_*in*_	*GV*_*in*_	*GV*_*in*_
Ref.^29^	2	4	9	1	16	$\frac{1 + 3D}{{(1 - D)}}$	$\frac{(G + 1)}{{2G}}V_{in}$	*GV*_*in*_	*GV*_*in*_
Ref.^31^	2	3	2	3	10	$\frac{D}{{(1 - D)^{2} }}$	$\frac{G}{D}V_{in}$	$\frac{G}{D}V_{in}$	*GV*_*in*_
Ref.^32^	1	4	3	6	14	$\frac{3D}{{(1 - D)}}$	$\frac{(G + 3)}{3}V_{in}$	$\frac{(G + 3)}{3}V_{in}$	*GV*_*in*_
Ref.^33^	2	2	2	2	8	$\frac{2}{(1 - D)}$	$\frac{G}{2}V_{in}$	*GV*_*in*_	*GV*_*in*_
Two cells proposed	1	2	4	3	10	$\frac{(4 - 3D)}{{(1 - D)}}$	$(G - 1)V_{in}$	$(G - 1)V_{in}$	*GV*_*in*_

Figure 20 illustrates the variation of voltage gain when the duty cycle is varied, the proposed converter has the highest voltage gain for *D* ≤ 0.5. For *D* > 0.5, the converter in^29^ has the highest voltage gain value. However, the proposed topology has a total number of components of 10 components, but the converter in^29^ has 16 components which increases the cost and complexity of the system and decreases the efficiency. Also, the proposed converter has higher voltage gain for duty cycle varies from 0.5 to 0.67 than converters in^31–33^, and the conventional one. The normalized maximum switch voltage stress for each converter is recorded in Table 3 and shown in Fig. 21. Converter in^31^ has the highest normalized switch voltage stress, while converter in^29^ has the lowest normalized voltage stress, and approximately constant for all voltage gain values. The introduced converter has modest normalized voltage stress across the main switch. The normalized maximum output diode voltage stress for each converter is documented in Table 3 and shown in Fig. 22. The suggested converter has the lower normalized output diode voltage stress except the converter in^32^ that has the lowest value. However, the converter in^32^ has total number of components of 14 components which greater than the proposed converter that decreases the efficiency and increases the cost and the volume of the converter. From this comparative analysis, it is proved that the suggested converter has significant features in comparison to the modern topologies.

## Conclusion

A new design of compact circuit that converts a low-level dc voltage to a high-level dc voltage has been proposed in this paper. It has a single switch and fewer passive components compared with recent boost converters. The new converter gets a high voltage gain at modest duty cycle, low switching losses, good efficiency, and it can be extended to get higher voltage gains by increasing the cascading additional cells. The suggested converter has been examined during open and closed loop process, and ensures a good control performance under reference voltage, input voltage, and load changes. Experimental results under different operating situations prove the usefulness of the new converter. In addition, the comparison between the new two-cell boost converter and other recent topologies has been presented. The proposed converter has the greatest voltage gain for duty cycle values D ≤ 0.5 with a total number of 10 components, which decreases the cost and complexity of the converter and increases the efficiency.

## Data Availability

All data generated or analyzed during this study are included in this published article.
